# Physical activity and hypertension amongst HIV-positive and HIV-negative populations in rural South Africa

**DOI:** 10.1038/s41440-026-02652-2

**Published:** 2026-05-07

**Authors:** Benjamin Zebita Mayasi, Peter Asaga Mac

**Affiliations:** 1 School of Public Health, University of the Witwatersrand, Johannesburg, South Africa; 2 Institute for Infection Prevention and Control, Medical Center, Faculty of Medicine, University of Freiburg, Breisacher Str. 115B, Freiburg, 79106 Germany

**Keywords:** HIV/AIDSHypertension, Physical Activity, South Africa, Cardiovascular Disease, Sub-Saharan Africa

## Abstract

Hypertension represents a major public health challenge in sub-Saharan Africa, characterised by substantial underdiagnosis and inadequate management. This secondary analysis of cross-sectional survey data examined the association between physical activity patterns and hypertension prevalence amongst HIV-positive and HIV-negative populations in rural South African communities. Data were drawn from the ongoing Agincourt Health and Socio-Demographic Surveillance System Site (AHDSS), collected between August 2022 and May 2023, involving 4,436 participants aged ≥ 15 years. Physical activity was assessed using the Global Physical Activity Questionnaire (GPAQ) within the WHO STEPwise framework, and blood pressure was measured using an automated digital device (OMRON R6 wrist monitor). Statistical analyses employed chi-squared tests, two-sample *t* tests, and multivariable logistic regression models adjusting for demographic, anthropometric, metabolic, and behavioural confounders. Moderate physical activity was associated with lower odds of hypertension irrespective of HIV serostatus, supporting the integration of physical activity counselling into HIV care and primary healthcare services in resource-limited settings (adjusted OR = 0.74, 95% CI: 0.56–0.99, p = 0.043). Hypertension prevalence was 40.4% overall. HIV-positive males demonstrated higher prevalence (60.2%) compared with HIV-negative males (46.9%, p < 0.001). HIV-positive status was independently associated with increased odds of hypertension (adjusted OR = 1.45, 95% CI: 1.18–1.78). Age, male sex, obesity, elevated waist-hip ratio, and current alcohol consumption showed significant positive associations with hypertension. Moderate physical activity provides protective benefits against hypertension irrespective of HIV serostatus, supporting the integration of physical activity counselling into HIV care and primary healthcare services in resource-limited settings.

**Figure:**
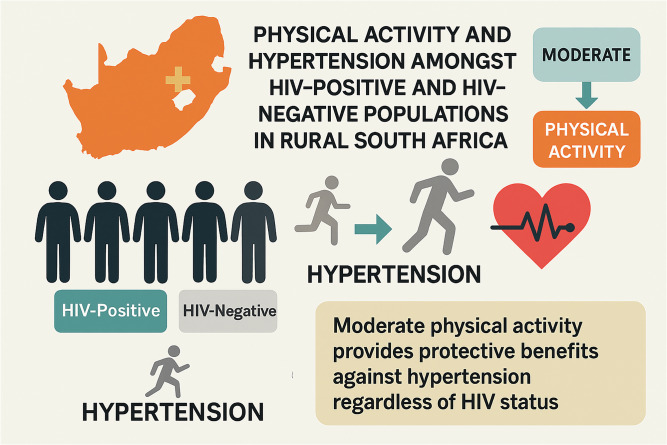
Visual summary of key findings. Physical activity and hypertension amongst HIV-positive and HIV-negative populations in rural South Africa: moderate physical activity provides protective benefits against hypertension regardless of HIV status

Visual summary of key findings. Physical activity and hypertension amongst HIV-positive and HIV-negative populations in rural South Africa: moderate physical activity provides protective benefits against hypertension regardless of HIV status

## Abstract (web summary)

Visual summary of key findings. Physical activity and hypertension amongst HIV-positive and HIV-negative populations in rural South Africa: moderate physical activity provides protective benefits against hypertension regardless of HIV status.

## Introduction

Hypertension, defined as persistently elevated arterial blood pressure, constitutes a leading modifiable risk factor for cardiovascular morbidity and mortality globally [1]. In sub-Saharan Africa, the burden of hypertension is escalating rapidly owing to urbanisation, epidemiological transition, and lifestyle changes, with prevalence estimates ranging from 20 to 50% across populations [1, 2]. Within South Africa, hypertension is an established risk factor for myocardial infarction, cerebrovascular accidents, chronic kidney disease, and retinopathy, and remains inadequately diagnosed and treated across communities [2, 3].

Hypertension frequently coexists with additional risk factors for chronic diseases of lifestyle, including diabetes mellitus, obesity, and physical inactivity. These interrelationships between hypertension and other chronic disease risk factors, combined with the diverse organ systems affected by uncontrolled hypertension, generate a complex clinical picture with substantial population health implications [4–7].

Physical activity has demonstrated consistent protective effects against hypertension through multiple physiological mechanisms, including enhancement of endothelial function, reduction of systemic vascular resistance, attenuation of sympathetic nervous system activity, and improvement of insulin sensitivity [3, 4, 8]. In the context of HIV infection, these mechanisms assume particular importance, as HIV-positive individuals experience chronic immune activation, systemic inflammation, and endothelial dysfunction that independently promote hypertension [9–11]. Furthermore, antiretroviral therapy (ART) may induce metabolic disturbances including lipodystrophy and insulin resistance, which further elevate cardiovascular risk [12, 13]. Physical activity may attenuate these pathological processes, though population-based evidence examining physical activity–hypertension associations stratified by HIV serostatus remains limited, particularly in rural African settings [5, 14, 15].

Whilst several large-scale studies have examined HIV–hypertension associations across sub-Saharan Africa, including the H3Africa AWI-Gen study [5], meta-analyses by Bigna et al. [16] and Davis et al. [17], and regional investigations in East and Southern Africa [18–24], few have incorporated domain-specific physical activity analysis using metabolic equivalent of task (MET)-based quantification. Understanding these associations in rural populations, where physical activity patterns differ substantially from urban settings, is essential for developing context-appropriate preventive strategies.

We hypothesised that higher levels of physical activity would be inversely associated with hypertension prevalence, and that this protective association would persist regardless of HIV serostatus. This secondary analysis of cross-sectional survey data therefore examined physical activity patterns and their association with hypertension prevalence amongst HIV-positive and HIV-negative populations in rural South Africa, utilising data from the Agincourt Health and Socio-Demographic Surveillance System.

## Methods

### Ethical considerations

The primary study received approval from the University of the Witwatersrand Human Research Ethics Committee (HREC clearance certificate number M10458) and the Mpumalanga Provincial Research and Ethics Committee. This secondary analysis was approved by the University of the Witwatersrand Human Research Ethics Committee (clearance certificate number M140917). Permission to utilise the data was obtained from the Agincourt Health and Socio-Demographic Surveillance Site. Written informed consent was obtained from all participants aged ≥ 18 years. For participants aged 15–17 years, both parental written consent and participant verbal assent were obtained. The study was conducted in accordance with the Declaration of Helsinki.

### Study design and setting

We conducted a secondary analysis of cross-sectional survey data from the Agincourt Health and Socio-Demographic Surveillance System Site (AHDSS), collected over 10 months from August 2022 to May 2023. The AHDSS monitors approximately 90,000 individuals residing in 15,500 households across 27 villages and is operated by the Medical Research Council (MRC)/Wits Rural Public Health and Health Transitions Research Unit. The surveillance site is situated in the Bushbuckridge sub-district of Ehlanzeni in Mpumalanga Province, northeastern South Africa. The AHDSS has recorded vital events, migration patterns, marital status, educational attainment, employment status, and socioeconomic indicators within this population since 1992 [25].

### Study population and sampling

The study population comprised all individuals participating in the AHDSS survey conducted between August 2022 and May 2023, totalling 4436 participants (1716 males and 2720 females, comprising both HIV-positive and HIV-negative individuals). Inclusion criteria specified age ≥ 15 years and permanent residence status according to the 2009 census round. The survey incorporated an oversample of 280 adults aged > 50 years from a previous investigation (the 2006 Study on Global Ageing and Adult Health, SAGE).

The primary survey utilised a sampling frame of 7662 individuals from a population of 34,413 based on the 2009 HDSS census round. All sampled individuals were visited by trained field interviewers proficient in the research protocol, HIV counselling, and dried blood spot collection procedures, with up to three home visits attempted. Sampling weights were not available for this secondary analysis; accordingly, prevalence estimates should be interpreted in the context of the sampling design rather than as population-representative figures.

### Data collection procedures

Home interviews (approximately 45 min duration) incorporated a chronic disease risk factor questionnaire, anthropometric measurements including height, weight, waist and hip circumference, blood pressure assessment, point-of-care lipid and glucose analysis, and dried blood spot collections for HIV testing.

### Physical activity assessment

Physical activity was assessed using the Global Physical Activity Questionnaire (GPAQ), a validated instrument developed by the World Health Organization for population surveillance within the WHO STEPwise approach to chronic disease risk factor surveillance [26, 27]. The GPAQ captures self-reported physical activity across three domains: occupational (vigorous and moderate), travel-related (walking or cycling), and recreational (vigorous), over a typical week. Metabolic equivalent of task (MET) values were assigned as follows: vigorous activities = 8.0 METs, moderate activities and travel-related activities = 4.0 METs [26] (Supplementary Table S1). Total physical activity volume was calculated as MET-minutes per week by multiplying the MET value by duration (minutes) and frequency (days per week) for each domain. Participants were subsequently stratified into quintiles of total MET-minutes/week for dose–response analysis (Supplementary Table S2). Activity levels were additionally classified as low (<600 MET-minutes/week), moderate (600–2999 MET-minutes/week), and high (≥3,000 MET-minutes/week) following WHO recommendations [26, 28].

### Blood pressure measurement and classification

Blood pressure was measured thrice on the right arm of seated participants using an automated digital recording instrument (OMRON R6 Wrist Blood Pressure Monitor, model HEM-6221-E; Omron Healthcare Co., Kyoto, Japan). Participants were seated for a minimum of five minutes prior to measurement, with the wrist positioned at heart level as per manufacturer instructions. Measurements were conducted in a quiet environment at ambient room temperature (20–25 °C) by trained field workers who had completed standardised blood pressure measurement training. The cuff size accommodated wrist circumferences of 13.5–21.5 cm. The mean of the second and third systolic blood pressure (SBP) and diastolic blood pressure (DBP) measurements was utilised for analysis. Full measurement protocol details, including device specifications, field worker training, and known limitations of wrist-based devices, are provided in Supplementary Section 2.

Blood pressure was classified following World Health Organization standards [29, 30]. Normotension was defined as SBP < 120 mmHg and DBP < 80 mmHg. Elevated blood pressure was defined as SBP 120–139 mmHg and/or DBP 80–89 mmHg. Hypertension was defined as SBP ≥ 140 mmHg and/or DBP ≥ 90 mmHg, or current use of antihypertensive medication. Participants were classified as hypertensive or non-hypertensive for the primary analysis.

### HIV status ascertainment

Informed consent was obtained prior to HIV testing. Dried blood spots were analysed using screening assay Vironostika Uniform 11 (Biomerieux, France), with positive results confirmed using the SD Bioline HIV ELISA test. In cases of discordant results, a third assay was performed to determine final serostatus following WHO criteria [8].

### Assessment of potential confounders

Anthropometric measurements including BMI (weight/height²) and waist-to-hip ratio (WHR) were obtained whilst participants wore light clothing without footwear. Elevated blood glucose and lipid concentrations were measured at each visit. Demographic variables including sex, age, marital status, and educational attainment, alongside behavioural factors including smoking status and alcohol consumption, and medical history including chronic diseases, were assessed via questionnaire.

### Statistical analysis

All data cleaning and statistical analyses were performed using Stata version 13 (StataCorp, College Station, Texas, USA). The initial sample comprised 4504 participants; 68 (1.5%) were excluded owing to >20% missing data on key variables, yielding a final analytical sample of 4436. Comparison of included and excluded participants revealed no statistically significant differences in age, sex, HIV status, or BMI (Supplementary Table S3). Little’s MCAR test (p = 0.23) suggested data were missing completely at random, supporting complete case analysis.

Smoking status and alcohol consumption were categorised as never, past, or current. Body mass index was categorised as underweight (<18.5 kg/m²), normal weight (18.5–24.9 kg/m²), overweight (25.0–29.9 kg/m²), and obese (≥30.0 kg/m²). Educational attainment was categorised as none, primary, secondary, or tertiary.

Descriptive statistics are presented as frequencies and percentages for categorical variables and means with standard deviations for continuous variables. Chi-squared tests examined associations between categorical variables, and two-sample t-tests compared continuous variables between groups. Bivariate and multivariable logistic regression models examined associations between physical activity and hypertension. Covariates demonstrating p < 0.20 in bivariate analysis, plus age and sex regardless of p-value, were included in multivariable models. Multicollinearity was assessed using variance inflation factors (maximum VIF = 4.2). Model fit was evaluated using the Hosmer–Lemeshow goodness-of-fit test (p = 0.42) and area under the receiver operating characteristic curve (AUC = 0.78, 95% CI: 0.76–0.79). Interaction terms between HIV status and physical activity were tested but did not improve model fit (all p > 0.10). The precision of effect estimates was assessed through 95% confidence intervals. Detailed statistical methods, power calculations, and sensitivity analyses are presented in the Supplementary Materials.

## Results

### Baseline characteristics

Table 1 presents baseline characteristics stratified by hypertension status. Amongst participants with hypertension (n = 1793), 775 (43.2%) were male and 1018 (56.8%) were female. Amongst non-hypertensive participants (n = 2643), 932 (35.3%) were male and 1711 (64.7%) were female, with a statistically significant sex distribution differences (p < 0.001). Mean age was significantly higher amongst hypertensive participants (51.8 ± 17.2 years) compared with non-hypertensive participants (36.1 ± 16.4 years, p < 0.001). HIV-positive status was observed in 25.0% (n = 1108) of the study population.

**Table 1:** Baseline characteristics of study participants by hypertension status

Characteristic	Total (N = 4436)	Hypertension (n = 1793)	Non-hypertensive (n = 2643)	P-value
	n (%)	n (%)	n (%)	
**Sex**				<0.001
Male	1716 (38.7)	775 (43.2)	932 (35.3)	
Female	2720 (61.3)	1018 (56.8)	1711 (64.7)	
**Age (years), mean ± SD**	42.3 ± 18.6	51.8 ± 17.2	36.1 ± 16.4	<0.001
**BMI (kg/m²), mean ± SD**	24.8 ± 6.2	26.4 ± 6.8	23.7 ± 5.6	<0.001
**HIV Status**				<0.001
Negative	3327 (75.0)	1434 (80.0)	1893 (71.6)	
Positive	1108 (25.0)	359 (20.0)	750 (28.4)	

P values from chi-squared tests for categorical variables and independent-samples t tests for continuous variables. Non-hypertensive defined as SBP < 140 mmHg and DBP < 90 mmHg without antihypertensive medication

*BMI* body mass index, *SD* standard deviation, *HTN* hypertension

### Socio-demographic characteristics

Table 2 presents socio-demographic characteristics of the study population (N = 4436). Females comprised 61.3% (n = 2720) and males 38.7% (n = 1716) of participants. Hypertension prevalence was significantly higher amongst males (45.4%) compared with females (37.9%) (p < 0.001). Hypertension prevalence increased progressively with age, with the highest prevalence observed in participants aged ≥70 years (62.3%, p < 0.001). Although prevalence increased with age overall, the proportion observed in the youngest age category in the age-stratified table should be interpreted cautiously, as single-occasion blood pressure measurement and transient physiological elevations may overestimate hypertension in younger participants.

**Table 2:** Socio-demographic characteristics and hypertension prevalence

Variable	Total N	Total %	HTN n	HTN %	*P* value	P-trend
**Sex**					<0.001	
Male	1716	38.7	779	45.4		
Female	2720	61.3	1031	37.9		
**Age group (years)**					<0.001	
15–19	502	11.3	122	24.3		
20–29	858	19.3	172	20.1		
30–39	811	18.3	270	33.3		
40–49	645	14.5	318	49.3		
50–59	553	12.5	299	54.1		
60–69	390	8.8	237	60.8		
≥70	521	11.7	325	62.3		<0.001
**Educational Attainment**					<0.001	
None	1063	24.0	591	55.6		
Primary	775	17.5	340	43.9		
Secondary	2439	55.0	810	33.2		
Tertiary	159	3.6	52	32.7		<0.001
**BMI Category (kg/m²)**					<0.001	
Underweight (<18.5)	312	7.4	104	33.3		
Normal (18.5–24.9)	2053	48.5	705	34.3		
Overweight (25.0–29.9)	1023	24.2	480	46.9		
Obese (≥30.0)	844	19.9	450	53.3		<0.001
**Alcohol Consumption**					<0.001	
Never	2512	56.6	960	38.2		
Past	1049	23.7	431	41.1		
Current	874	19.7	420	48.1		

Percentages calculated as column percentages for Total % and as prevalence (proportion with hypertension within each stratum) for HTN %. P values from chi-squared tests. P-trend calculated using the Cochran–Armitage test for trend across ordered categories

*HTN* hypertension, *BMI* body mass index

Educational attainment distribution revealed 24.0% (n = 1063) with no formal education, 17.5% (n = 775) with primary education, 55.0% (n = 2439) with secondary education, and 3.6% (n = 159) with tertiary education. Hypertension prevalence was highest amongst those without formal education (55.6%, p < 0.001). Body mass index distribution showed 7.4% (n = 312) underweight, 48.5% (n = 2053) normal weight, 24.2% (n = 1023) overweight, and 19.9% (n = 844) obese. Hypertension prevalence increased progressively with BMI category, reaching 53.3% amongst obese participants (p for trend <0.001). Current alcohol consumption was associated with 48.1% hypertension prevalence compared with 38.2% amongst never-consumers (p < 0.001).

### Physical activity patterns by HIV status

**Fig. 1:**
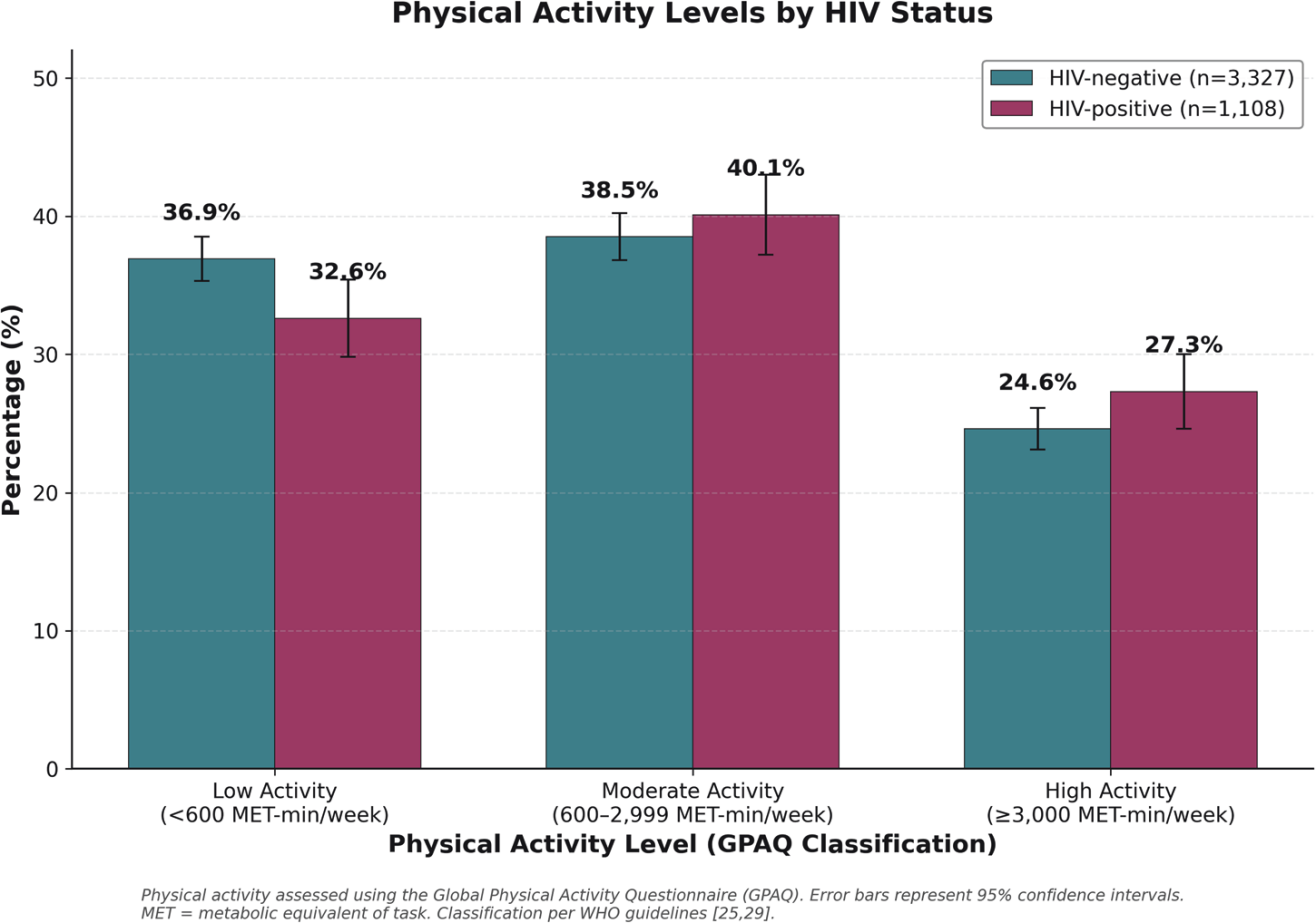
Physical activity levels by HIV status. Bar chart showing the distribution of low, moderate, and high physical activity levels amongst HIV-negative and HIV-positive participants. Error bars represent 95% confidence intervals

Table 3 presents physical activity patterns stratified by HIV serostatus (Fig. 1). Amongst 4435 participants with HIV results, 75.0% (n = 3327) were HIV-negative and 25.0% (n = 1108) were HIV-positive. Vigorous recreational activities showed 80.3% HIV-negative participants amongst active individuals and 26.2% HIV-positive amongst inactive participants, indicating a higher inactivity rate in vigorous recreational pursuits amongst HIV-positive participants.

**Table 3:** Physical activity patterns stratified by HIV serostatus

Physical activity domain	Activity status	Total N	HIV-Negative n	HIV-Negative %	HIV-Positive n	HIV-Positive %	P value
Overall Sample	—	4435	3327	75.0	1108	25.0	—
Vigorous Occupational	Active	2847	2100	73.8	747	26.2	0.007
	Inactive	1588	1227	77.3	361	22.7	
Moderate Occupational	Active	2121	1576	74.3	545	25.7	0.276
	Inactive	2314	1751	75.7	563	24.3	
Travel-Related	Active	3124	2327	74.5	797	25.5	0.198
	Inactive	1311	1000	76.3	311	23.7	
Vigorous Recreational	Active	817	656	80.3	161	19.7	<0.001
	Inactive	3618	2671	73.8	947	26.2	

Percentages represent the proportion of each activity status group by HIV serostatus. Active status defined as engaging in the specified activity domain during a typical week; Inactive defined as not engaging in the activity. Physical activity assessed using the GPAQ within the WHO STEPwise framework [26]. P values from chi-squared tests comparing HIV-negative and HIV-positive distributions within each domain

*HIV* − HIV-negative, *HIV* + HIV-positive, *GPAQ* Global Physical Activity Questionnaire, *MET* metabolic equivalent of task

### Hypertension prevalence by HIV status

**Fig. 2:**
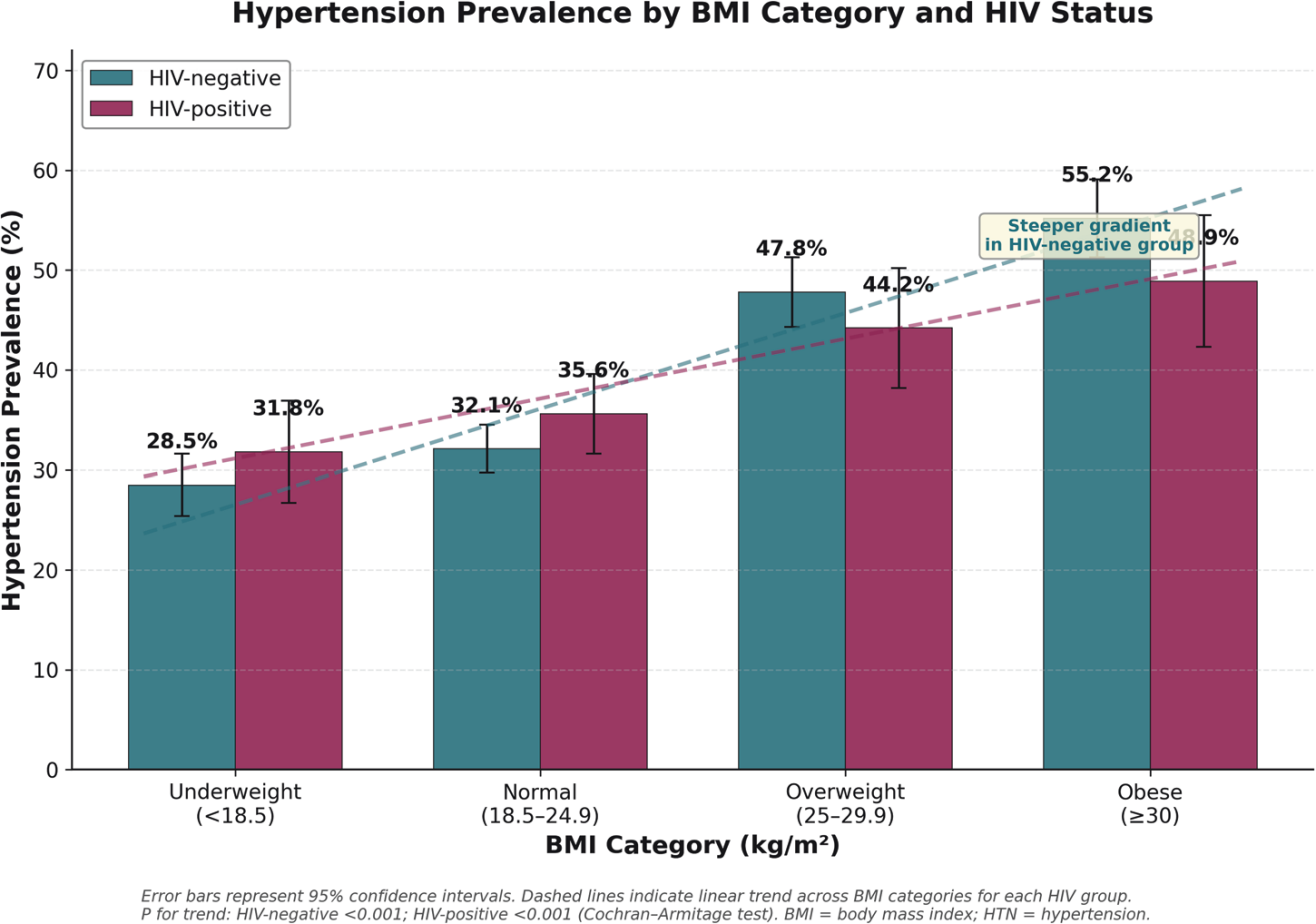
Hypertension prevalence by BMI category and HIV status. Grouped bar chart demonstrating hypertension prevalence across body mass index categories, stratified by HIV serostatus. Error bars represent 95% confidence intervals. Dashed trend lines illustrate the gradient in hypertension prevalence across BMI categories for each HIV group

Table 4 presents hypertension prevalence stratified by HIV serostatus and demographic characteristics. HIV-positive males showed highest hypertension prevalence (60.2%) compared with HIV-negative males (46.9%, p < 0.001). Figure 2 illustrates hypertension prevalence by BMI category and HIV status, revealed a steeper gradient in hypertension prevalence across BMI categories amongst HIV-negative participants. Statistically significant associations (p < 0.05) were observed between hypertension and sex, age group, BMI, alcohol consumption, and smoking status in both HIV-positive and HIV-negative groups.

**Table 4:** Hypertension prevalence stratified by HIV serostatus and demographic characteristics

Characteristic	HIV − n (%)	HIV − HTN %	HIV + n (%)	HIV + HTN %	Overall HTN %	P value	P-trend
Sex						<0.001	
Male	1355 (79.0)	46.9	361 (21.0)	60.2	49.7		
Female	1972 (72.5)	41.1	748 (27.5)	29.6	37.9		
Age (years)						<0.001	
15–19	483 (96.2)	24.3	19 (3.8)	26.3	24.3		
20–24	425 (91.4)	18.6	40 (8.6)	15.0	18.3		
25–29	329 (83.7)	21.0	64 (16.3)	23.4	21.4		
30–34	318 (76.6)	30.5	97 (23.4)	26.8	29.6		
35–39	283 (71.5)	42.0	113 (28.5)	20.4	35.9		
40–44	194 (64.0)	48.5	109 (36.0)	38.5	44.9		
45–49	235 (68.7)	52.8	107 (31.3)	47.7	51.2		
50–54	222 (70.9)	54.5	91 (29.1)	51.6	53.7		
55–59	169 (70.4)	57.4	71 (29.6)	52.1	55.8		
60–64	141 (71.9)	58.9	55 (28.1)	56.4	58.2		
65–69	142 (73.2)	61.3	52 (26.8)	59.6	60.8		
≥70	386 (74.1)	63.1	135 (25.9)	60.7	62.3		<0.001
BMI (kg/m²)						<0.001	
Underweight (<18.5)	230 (73.7)	28.5	82 (26.3)	31.8	33.3		
Normal (18.5–24.9)	1506 (73.4)	32.1	547 (26.6)	35.6	34.3		
Overweight (25–29.9)	762 (74.5)	47.8	261 (25.5)	44.2	46.9		
Obese (≥30)	625 (74.1)	55.2	219 (25.9)	48.9	53.3		<0.001
Education						<0.001	
None	801 (75.4)	55.6	262 (24.6)	55.3	55.6		
Primary	569 (73.4)	43.9	206 (26.6)	44.2	43.9		
Secondary	1827 (74.9)	33.2	612 (25.1)	33.5	33.2		
Tertiary	130 (81.8)	32.3	29 (18.2)	34.5	32.7		<0.001

Percentages in n (%) columns represent the distribution of HIV serostatus within each demographic stratum. HTN % columns show the percentage of participants with hypertension within each HIV-serostatus subgroup. Overall HTN % shows combined hypertension prevalence across both HIV groups within each stratum. P-values from chi-squared tests. P-trend calculated using the Cochran–Armitage test for trend across ordered categories. Note: HIV-positive female HTN prevalence (29.6%) represents age-unadjusted prevalence across all age strata within this subgroup; the lower value compared with HIV-negative females (41.1%) reflects the younger age distribution of HIV-positive women in this cohort

*HTN* hypertension, *HIV −* HIV-negative, *HIV +* HIV-positive, *BMI* body mass index, *WHR* waist-to-hip ratio

### Physical activity and hypertension: regression analysis

Table 5 presents univariate and multivariate analyses of physical activity domains and hypertension. In univariate analysis, moderate occupational activity showed a statistically significant positive association (OR = 1.37, 95% CI: 1.14–1.65, p = 0.001), which was attenuated and became non-significant after multivariate adjustment (OR = 1.10, 95% CI: 0.89–1.36, p = 0.368). This reversal in direction is consistent with confounding, likely by age and sex: older and male participants, who had higher hypertension prevalence, were more likely to engage in moderate occupational activity. After adjustment for these confounders, the underlying protective effect of moderate physical activity became apparent.

**Table 5:** Univariate and multivariate logistic regression analysis of physical activity domains and hypertension

Physical activity domain	Univariate OR	95% CI	P value	Adjusted^†^ OR	95% CI	P value
**Vigorous Occupational Activity**	1.04	0.89–1.20	0.626	0.98	0.81–1.18	0.823
**Moderate Occupational Activity**	1.37	1.14–1.65	0.001	1.10	0.89–1.36	0.368
**Travel-Related Physical Activity**	1.08	0.87–1.34	0.468	1.05	0.82–1.35	0.686
**Vigorous Recreational Activity**	1.72	1.41–2.09	0.001	1.87	1.13–3.08	0.013
**Total Physical Activity Volume**	0.63	0.48–0.82	0.001	0.44	0.23–0.85	0.015
**Total Physical Activity Quintiles**			0.001			0.137^‡^
Quintile 1 (lowest)	1.00	Reference	—	1.00	Reference	—
Quintile 2	0.89	0.70–1.14	0.354	0.92	0.70–1.21	0.558
Quintile 3	0.76	0.59–0.98	0.034	0.85	0.63–1.14	0.273
Quintile 4	0.68	0.52–0.88	0.003	0.79	0.58–1.08	0.140
Quintile 5 (highest)	0.61	0.47–0.80	<0.001	0.73	0.53–1.00	0.051

Reference category for all binary physical activity variables is “Inactive”. †Multivariate models adjusted for age, sex, BMI, HIV status, alcohol consumption, smoking status, and educational attainment. ‡P-value for trend across quintiles (Cochran–Armitage test). Total physical activity volume (MET-minutes/week) represents the combined metabolic equivalent of task minutes per week across all activity domains (occupational vigorous/moderate, travel-related, and recreational), calculated as: MET value × duration (minutes) × frequency (days/week) [26]

*OR* odds ratio, *CI* confidence interval, *MET* metabolic equivalent of task, *GPAQ* Global Physical Activity Questionnaire.

Total physical activity volume (MET-minutes/week) revealed a protective association, with univariate OR = 0.63 (95% CI: 0.48–0.82, p = 0.001) and multivariate OR = 0.44 (95% CI: 0.23–0.85, p = 0.015), indicating a protective effect against hypertension. Quintile analysis showed a trend towards protection in higher activity quintiles in univariate analysis (p for trend=0.001), which was attenuated in multivariate analysis (p for trend=0.137).

### Multivariable analysis including confounders

**Fig. 3:**
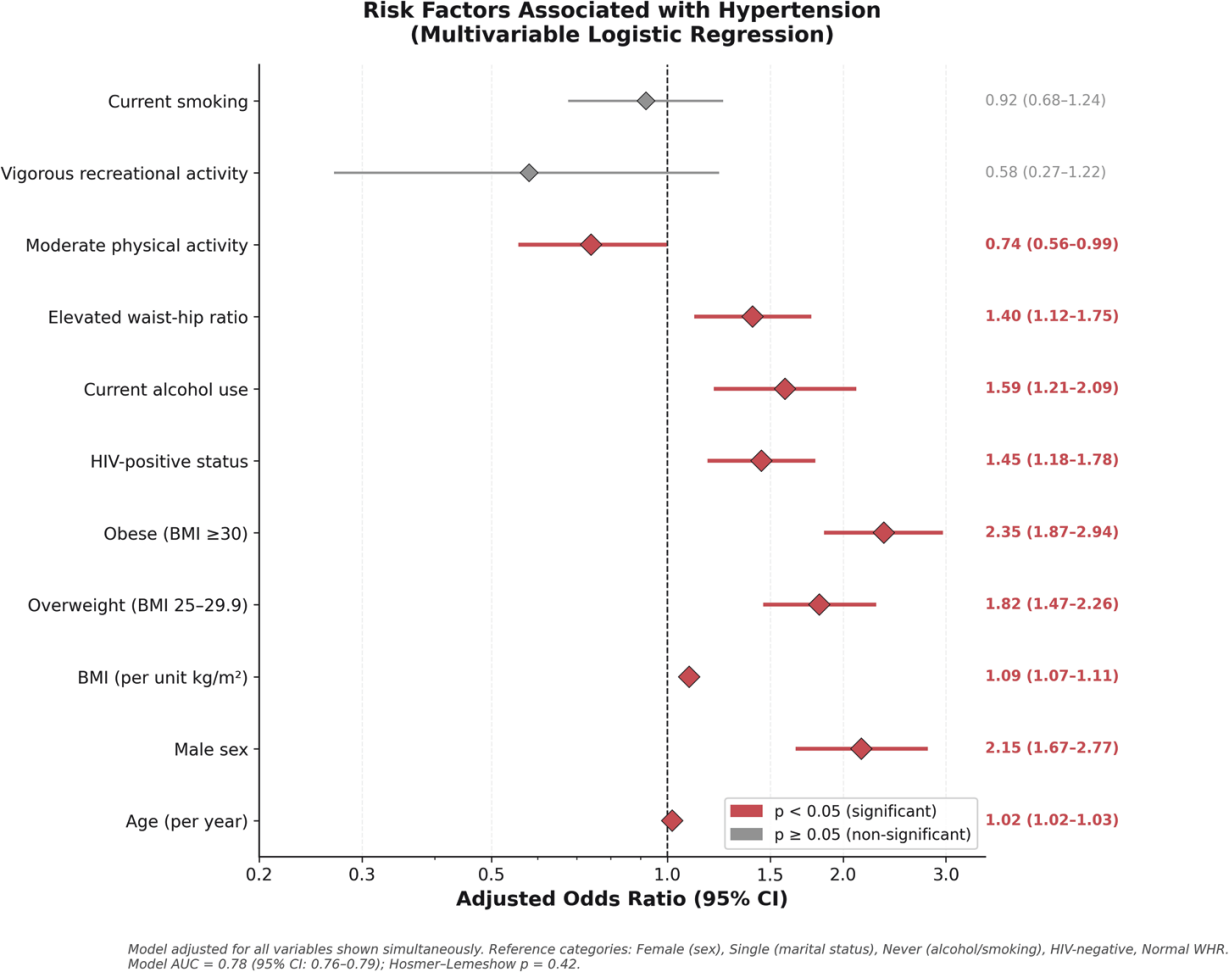
Risk factors associated with hypertension (multivariable logistic regression). Forest plot displaying adjusted odds ratios with 95% confidence intervals for each covariate in the final multivariable model. Red diamonds indicate statistically significant associations (p < 0.05); grey diamonds indicate non-significant associations. The dashed vertical line represents the null value (OR = 1.0)

Table 6 presents comprehensive multivariable analysis incorporating all potential confounders (Fig. 3). Moderate physical activity was association (adjusted OR = 0.74, 95% CI: 0.56–0.99, p = 0.043), whilst vigorous recreational activities showed a non-significant protective trend (adjusted OR = 0.58, 95% CI: 0.27–1.22, p = 0.151) after full covariate adjustment.

**Table 6:** Comprehensive multivariable logistic regression analysis of factors associated with hypertension

Variable	Univariate OR	95% CI	P value	Adjusted OR	95% CI	P value
**Physical Activity**						
Moderate physical activity	1.37	1.14–1.65	0.001	0.74	0.56–0.99	0.043
Vigorous recreational activity	1.72	1.41–2.09	0.001	0.58	0.27–1.22	0.151
**Demographics**						
Male sex	1.81	1.53–2.14	<0.001	2.15	1.67–2.77	<0.001
Age (per year increase)	1.05	1.04–1.05	<0.001	1.02	1.02–1.03	<0.001
**Marital Status (vs. Single)**						
In relationship	1.42	1.13–1.79	0.003	1.21	0.88–1.65	0.235
Unknown	2.09	1.72–2.54	<0.001	1.09	0.82–1.47	0.534
**Education (vs. None)**						
Primary	0.58	0.47–0.71	<0.001	0.92	0.70–1.20	0.530
Secondary	0.34	0.29–0.40	<0.001	0.84	0.66–1.07	0.156
Tertiary	0.36	0.25–0.53	<0.001	0.78	0.48–1.27	0.317
**Anthropometric Measures**						
BMI (per kg/m² increase)	1.12	1.10–1.13	<0.001	1.09	1.07–1.11	<0.001
Overweight (BMI 25–29.9)	1.69	1.43–2.00	<0.001	1.82	1.47–2.26	<0.001
Obese (BMI ≥ 30)	2.19	1.83–2.63	<0.001	2.35	1.87–2.94	<0.001
Elevated WHR	2.24	1.89–2.65	<0.001	1.40	1.12–1.75	0.004
**Lifestyle Factors**						
Alcohol: Past (vs. Never)	1.13	0.96–1.34	0.148	1.00	0.79–1.24	0.943
Alcohol: Current (vs. Never)	1.51	1.26–1.80	<0.001	1.59	1.21–2.09	0.001
Smoking: Past (vs. Never)	1.18	0.94–1.48	0.159	0.89	0.66–1.20	0.433
Smoking: Current (vs. Never)	1.06	0.86–1.31	0.590	0.92	0.68–1.24	0.572
**HIV Status**						
HIV-positive (vs. HIV- negative)	0.61	0.51–0.73	<0.001	1.45	1.18–1.78	<0.001

Adjusted model includes all variables listed in the table simultaneously. Reference categories: Inactive (for physical activity), Female (for sex), Single (for marital status), None (for educational attainment), Never (for alcohol and smoking), HIV-negative (for HIV status), Normal WHR (for waist-to-hip ratio). Model diagnostics: AUC = 0.78 (95% CI: 0.76–0.79); Hosmer–Lemeshow test p = 0.42; Overall classification accuracy = 73.2%

*OR* odds ratio, *CI* confidence interval, *BMI* body mass index, *WHR* waist-to-hip ratio

Male sex group showed a positive association with hypertension (adjusted OR = 2.15, 95% CI: 1.67–2.77, p < 0.001). Age showed a positive association (adjusted OR = 1.02 per year, 95% CI: 1.02–1.03, p < 0.001). Body mass index revealed a positive association (adjusted OR = 1.09 per unit, 95% CI: 1.07–1.11, p < 0.001). Current alcohol consumption showed a strong positive association (adjusted OR = 1.59, 95% CI: 1.21–2.09, p = 0.001). Elevated waist-to-hip ratio was associated with increased odds of hypertension (adjusted OR = 1.40, 95% CI: 1.12–1.75, p = 0.004). HIV-positive status was independently associated with increased odds of hypertension (adjusted OR = 1.45, 95% CI: 1.18–1.78, p < 0.001).

## Discussion

This secondary analysis of cross-sectional data from the AHDSS indicates that moderate physical activity is independently associated with lower odds of hypertension in a rural South African population, irrespective of HIV serostatus. The study further confirms the high burden of hypertension (40.4%) and the independent association between HIV-positive status and hypertension in this setting.

An unexpected finding warrants careful interpretation: vigorous recreational physical activity was associated with positive hypertension in univariate analysis (OR = 1.72), which reversed to a non-significant protective trend in fully adjusted models (OR = 0.58). This reversal is consistent with confounding by socioeconomic status and health-seeking behaviour, whereby participants engaging in vigorous recreational activities may represent a higher socioeconomic stratum with greater hypertension awareness and diagnosis rates [5, 31]. After adjustment for age, BMI, and other cardiovascular risk factors, the physiological protective effects of vigorous activity emerged, although the wide confidence interval (0.27–1.22) and loss of statistical significance indicate insufficient statistical power for this subgroup or residual confounding. These findings should be interpreted cautiously, and future studies should incorporate objective physical activity measurement to disentangle these relationships.

### Physical activity and hypertension

Our findings revealed a statistically significant associations between physical activity and hypertension in rural South African communities, consistent with studies conducted in other world regions [3, 4, 32–34]. The protective influence of moderate physical activity on hypertension prevention (adjusted OR = 0.74, 95% CI: 0.56–0.99) supports physical activity promotion as a viable hypertension prevention strategy. Interestingly, vigorous recreational activities showed a non-significant association in fully adjusted models, possibly reflecting complex interactions with other health behaviours or reverse causation [32–36].

### Hypertension in rural South Africa

Hypertension prevalence in our study (40.4% overall) is consistent with contemporary population-based surveys in South Africa. The South African Demographic and Health Survey (SADHS 2016) reported age-standardised hypertension prevalence of 46% amongst adults aged ≥ 15 years [37], whilst Schutte et al. (2021) documented prevalence estimates of 35–55% across diverse South African settings [15]. Our findings align with these estimates and confirm hypertension as a substantial public health challenge in rural South Africa, particularly given the limited access to diagnosis and treatment in this setting.

Advanced age constitutes an established risk factor for cardiovascular disease [38, 39], consistent with our findings of progressive hypertension prevalence increases with age. Male sex was strongly associated with hypertension (adjusted OR = 2.15), consistent with established sex differences in cardiovascular risk.

### HIV and hypertension

The elevated hypertension prevalence amongst HIV-positive participants, particularly males (60.2% vs. 46.9% in HIV-negative males), likely reflects multiple interacting mechanisms: (1) HIV-induced chronic immune activation and systemic inflammation contribute to endothelial dysfunction and arterial stiffness [9–11]; (2) ART-related metabolic effects, including lipodystrophy and insulin resistance, further elevate cardiovascular risk [12, 13]; (3) HIV-associated nephropathy and drug-related nephrotoxicity may compromise renal function, activating the renin–angiotensin–aldosterone system [9, 18, 23]; and (4) sex-specific interactions between HIV infection, ART effects, and traditional cardiovascular risk factors may underlie the particularly elevated prevalence in HIV-positive males [7, 21, 24]. Our finding of 45% increased odds of hypertension amongst HIV-positive individuals aligns with meta-analyses across sub-Saharan Africa [16, 17, 40, 41], and comparable studies from Tanzania [18, 23], Uganda [19, 22], Kenya [42], and Malawi [42]. However, the absence of ART data in our study precludes definitive conclusions regarding the specific contribution of therapy-related versus infection-related mechanisms.

### Study limitations

This study has several limitations that warrant consideration. First, the cross-sectional design precludes causal inference, and observed associations may be influenced by reverse causation, particularly where behavioural changes follow a diagnosis of hypertension. Second, physical activity was assessed through self-report using the GPAQ, physical activity was self-reported, misclassification of activity levels is possible and may have attenuated the observed associations, which is susceptible to recall bias and social desirability bias, although systematic validation studies showed reasonable correlation with objective measures (r = 0.45–0.65 for moderate-to-vigorous activity) [26, 27]. A comprehensive assessment of potential biases is provided in Supplementary Section 7. Third, blood pressure was measured using a wrist-based device (OMRON R6, HEM-6221-E), which may produce readings that differ from validated upper-arm devices [43]. Wrist monitors are sensitive to positioning and may overestimate or underestimate blood pressure if the wrist is not maintained at heart level; however, our field protocol required standardised positioning. Furthermore, blood pressure was measured on a single occasion, which may lead to misclassification of some individuals; such error would probably attenuate rather than exaggerate the associations observed [43]. Sensitivity analyses using alternative blood pressure definitions and borderline reclassification yielded consistent results (Supplementary Table S4).

Fourth, although we adjusted for a broad range of covariates, residual confounding from unmeasured factors, including dietary patterns, psychosocial stress, and genetic predisposition, cannot be excluded. Fifth, the absence of antiretroviral therapy data (including regimen, duration, adherence, CD4 count, and viral load) limits our ability to disentangle the independent contributions of HIV infection from ART-related metabolic effects on hypertension risk. Sixth, sampling weights were not available for this secondary analysis, and the use of a 2009 sampling frame may introduce selection bias. Accordingly, prevalence estimates should be interpreted with caution and may not be generalisable to the broader South African population. Finally, the study population was drawn from a single rural South African setting, which may constrain generalisability to other contexts.

Notwithstanding these limitations, our primary finding of a protective association between moderate physical activity and hypertension was robust across multiple sensitivity analyses, including HIV-stratified models, exclusion of antihypertensive medication users, alternative blood pressure definitions, and borderline reclassification (Supplementary Table S5). These findings should therefore be interpreted as context-specific and hypothesis-generating rather than broadly generalisable to all South African or sub-Saharan African populations.

### Clinical and public health implications

Our findings support urgent integration of cardiovascular risk assessment into HIV care platforms [42, 44, 45]. The observed association between moderate physical activity and lower odds of hypertension, consistent across HIV serostatus, supports consideration of physical activity promotion as a cost-effective intervention strategy [28, 34]. Healthcare systems must prepare for the double burden of communicable and non-communicable diseases through task-shifting approaches and integrated care models [31, 44, 46, 47].

## Conclusions

Hypertension demonstrates high prevalence amongst HIV-positive and HIV-negative adults in rural South Africa, with HIV-positive status independently associated with increased odds of hypertension. Moderate physical activity provides significant protective benefits against hypertension irrespective of HIV serostatus. These findings support the integration of structured physical activity counselling into HIV care and primary healthcare platforms in resource-limited settings. Large-scale prospective cohort studies incorporating objective physical activity measurement and detailed ART data are needed to confirm the protective association observed in this cross-sectional analysis and to elucidate the mechanisms underlying HIV-associated hypertension.

## Supplementary information

Supplementary Materials

STROBE Checklist – Cross-sectional Study

## Data Availability

The data underlying this study are available from the Agincourt Health and Socio-Demographic Surveillance Site (www.agincourt.co.za) upon reasonable request and with appropriate ethical approval. Statistical analysis code (Stata do-files) is available from the corresponding author upon request.
