# Supplementary material for: Physical activity and hypertension amongst HIV-positive and HIV-negative populations in rural South Africa
Source: Hypertens Res. 2026 May 7;49(7):2128–40. doi: 10.1038/s41440-026-02652-2 (PMC13333498; doi:10.1038/s41440-026-02652-2)
Supplement: Supplementary file 1 — Supplementary Materials [file 41440_2026_2652_MOESM1_ESM.docx]

**Supplementary Materials**

*Physical Activity and Hypertension amongst HIV-Positive and HIV-Negative Populations in Rural South Africa*

# Supplementary Section 1. Physical Activity Instrument Details (GPAQ)

Physical activity was assessed using the Global Physical Activity Questionnaire (GPAQ), version 2, developed by the World Health Organization as part of the STEPwise approach to chronic disease risk factor surveillance [25]. The GPAQ captures information on physical activity in three domains and sedentary behaviour using 16 items, with a recall period of a typical week.

**Supplementary Table S1. GPAQ Domain Definitions and MET Assignments**

| **Domain** | **GPAQ Items** | **MET Value** | **Intensity** | **Example Activities** |
| --- | --- | --- | --- | --- |
| Vigorous Occupational | P1–P3 (days/week, hours, minutes) | 8.0 METs | Vigorous | Heavy lifting, digging, construction |
| Moderate Occupational | P4–P6 (days/week, hours, minutes) | 4.0 METs | Moderate | Brisk walking, carrying loads, farming |
| Travel-Related | P7–P9 (days/week, hours, minutes) | 4.0 METs | Moderate | Walking, cycling for transport |
| Vigorous Recreational | P10–P12 (days/week, hours, minutes) | 8.0 METs | Vigorous | Running, football, gymnasium |
| Moderate Recreational | P13–P15 (days/week, hours, minutes) | 4.0 METs | Moderate | Cycling, swimming, gardening |

*MET = metabolic equivalent of task. Total MET-minutes/week = Σ(MET value × minutes × days) across all domains. Activity level classification: Low (<600 MET-min/week), Moderate (600–2,999 MET-min/week), High (≥3,000 MET-min/week) per WHO recommendations [25,29].*

**Supplementary Table S2. MET-minutes/week Quintile Cut-Points**

| **Quintile** | **Range (MET-min/week)** | **n** | **% with HTN** | **Adjusted OR (95% CI)** |
| --- | --- | --- | --- | --- |
| Q1 (lowest) | 0–480 | 887 | 47.8 | 1.00 (Reference) |
| Q2 | 481–1,680 | 887 | 43.7 | 0.92 (0.70–1.21) |
| Q3 | 1,681–4,320 | 887 | 40.1 | 0.85 (0.63–1.14) |
| Q4 | 4,321–8,400 | 888 | 37.3 | 0.79 (0.58–1.08) |
| Q5 (highest) | >8,400 | 887 | 33.5 | 0.73 (0.53–1.00) |

*P for trend = 0.137 (adjusted model). Quintiles based on total MET-minutes/week across all GPAQ domains. HTN = hypertension; OR = odds ratio; CI = confidence interval.*

**Supplementary Section 2. Blood Pressure Measurement Protocol**

Blood pressure was measured using the OMRON R6 Wrist Blood Pressure Monitor (model HEM-6221-E; Omron Healthcare Co., Kyoto, Japan). The standardised field protocol was as follows:

Device specifications: Oscillometric wrist monitor; measurement range SBP 60–260 mmHg, DBP 40–199 mmHg; cuff circumference 13.5–21.5 cm; accuracy ±3 mmHg (pressure), ±5% (pulse); memory capacity 90 readings.

Measurement protocol: (1) Participants were seated comfortably with legs uncrossed for a minimum of five minutes prior to measurement. (2) The wrist cuff was applied to the left wrist at heart level, with the palm facing upward and the forearm supported on a flat surface. (3) Three sequential measurements were taken at one-minute intervals. (4) The mean of the second and third readings was used for analysis, discarding the first measurement to reduce white-coat effect. (5) Measurements were conducted in a quiet room at ambient temperature (20–25°C). (6) All field workers completed a standardised two-day blood pressure measurement training programme including practical competency assessment.

Limitations of wrist measurement: Wrist-based oscillometric devices may produce readings that differ from upper-arm measurements owing to differences in arterial anatomy and sensitivity to wrist positioning. Validation studies suggest mean differences of 1–5 mmHg between wrist and upper-arm devices, with greater variability when the wrist is not maintained at heart level [47]. Our field protocol required standardised wrist-at-heart-level positioning to minimise this source of error. Any residual measurement error from wrist-based assessment would be expected to be non-differential with respect to physical activity exposure, thereby attenuating rather than inflating observed associations.

# Supplementary Section 3. Missing Data Assessment

The initial sample comprised 4,504 participants. Following data cleaning procedures, 68 participants (1.5%) were excluded owing to missing data exceeding the 20% threshold on key variables, yielding a final analytical sample of 4,436 participants.

**Supplementary Table S3. Comparison of Included and Excluded Participants**

| **Characteristic** | **Included (n=4,436)** | **Excluded (n=68)** | **P-value** |
| --- | --- | --- | --- |
| Age, mean ± SD | 42.3 ± 18.6 | 43.8 ± 19.1 | 0.42 |
| Female, n (%) | 2,720 (61.3) | 44 (64.7) | 0.38 |
| HIV-positive, n (%) | 1,108 (25.0) | 15 (22.1) | 0.51 |
| BMI, mean ± SD | 24.8 ± 6.2 | 24.1 ± 5.9 | 0.35 |

*No statistically significant differences were observed between included and excluded participants, supporting the validity of complete case analysis. Little’s MCAR test: p=0.23, consistent with data missing completely at random.*

**Supplementary Section 4. Detailed Statistical Methods**

**4.1 Logistic Regression Modelling Strategy**

Multivariable logistic regression models were constructed using a systematic approach. Step 1: Univariate analyses identified potential confounders (p<0.20 threshold for inclusion). Step 2: Multivariable models incorporated all variables meeting the p<0.20 criterion, plus variables with established theoretical importance (age, sex) regardless of p-value. Step 3: Multicollinearity was assessed using variance inflation factors (VIF); variables with VIF >10 were examined for potential exclusion or combination. Maximum VIF in final models was 4.2, indicating acceptable collinearity levels. Step 4: Model fit was evaluated using the Hosmer–Lemeshow goodness-of-fit test (p>0.05 indicating acceptable fit), area under the receiver operating characteristic curve (AUC), and classification accuracy. Step 5: Interaction terms between HIV status and physical activity variables were tested but did not significantly improve model fit (all p>0.10) and were excluded from final models.

**4.2 Model Performance**

The final multivariable model achieved acceptable discrimination (AUC=0.78, 95% CI: 0.76–0.79) and calibration (Hosmer–Lemeshow p=0.42), with overall classification accuracy of 73.2%. Sensitivity was 64.1% and specificity was 79.4%.

**4.3 Confounding Assessment**

The reversal in direction of association for moderate physical activity between univariate (OR=1.37) and multivariate (adjusted OR=0.74) analyses is consistent with confounding by age and sex. Older participants and males were more likely to engage in moderate occupational physical activity and independently had higher hypertension prevalence. After adjustment, the underlying protective effect of moderate physical activity became apparent. Sensitivity analyses examining sequential covariate addition confirmed that age was the primary confounder responsible for this reversal.

**Supplementary Section 5. Sensitivity Analyses**

**5.1 Alternative Blood Pressure Definitions**

Sensitivity analyses examined whether associations remained consistent using alternative hypertension definitions:

Definition 1 (Primary): SBP ≥140 mmHg or DBP ≥90 mmHg or current antihypertensive medication use.

Definition 2: SBP ≥140 mmHg and/or DBP ≥90 mmHg (excluding medication users).

Definition 3: Elevated blood pressure/hypertension combined (SBP ≥120 mmHg or DBP ≥80 mmHg).

Primary findings remained consistent across definitions, with moderate physical activity showing protective associations (adjusted OR range: 0.71–0.78) and current alcohol consumption showing positive associations (adjusted OR range: 1.53–1.64) across all three definitions.

**Supplementary Table S4. Sensitivity Analysis: Moderate Physical Activity and Hypertension by Alternative Definitions**

| **BP Definition** | **HTN Prevalence** | **Adjusted OR (95% CI)** | **P-value** | **n** |
| --- | --- | --- | --- | --- |
| Definition 1 (primary) | 40.4% | 0.74 (0.56–0.99) | 0.043 | 4,436 |
| Definition 2 (no medication) | 37.2% | 0.73 (0.54–0.99) | 0.041 | 4,094 |
| Definition 3 (elevated + HTN) | 62.8% | 0.78 (0.61–1.01) | 0.058 | 4,436 |

*All models adjusted for age, sex, BMI, HIV status, alcohol consumption, smoking status, educational attainment, waist-to-hip ratio, and lipid profiles. BP = blood pressure; HTN = hypertension; OR = odds ratio; CI = confidence interval.*

**5.2 Stratified Analyses by HIV Status**

Separate multivariable models were constructed for HIV-positive and HIV-negative participants:

HIV-Negative Participants (n=3,327): Moderate physical activity adjusted OR=0.72, 95% CI: 0.53–0.98, p=0.037.

HIV-Positive Participants (n=1,108): Moderate physical activity adjusted OR=0.79, 95% CI: 0.48–1.29, p=0.342.

Formal interaction testing (HIV status × moderate physical activity) yielded p=0.68, indicating no statistically significant effect modification by HIV status, though reduced power in stratified analyses limits definitive conclusions.

**5.3 Exclusion of Medication Users**

A sensitivity analysis excluding participants reporting current antihypertensive medication use (n=342, 7.7%) yielded adjusted OR=0.73 (95% CI: 0.54–0.99, p=0.041), indicating robust findings independent of treatment status.

**5.4 Age-Stratified Analyses**

Younger adults (15–39 years, n=2,387): adjusted OR=0.68, 95% CI: 0.45–1.02, p=0.062.

Middle-aged adults (40–59 years, n=1,076): adjusted OR=0.71, 95% CI: 0.48–1.04, p=0.078.

Older adults (≥60 years, n=973): adjusted OR=0.82, 95% CI: 0.51–1.32, p=0.412.

Whilst point estimates suggested stronger protective associations amongst younger adults, confidence intervals overlapped substantially and interaction testing was non-significant (p=0.54).

**5.5 Borderline Blood Pressure Reclassification**

A sensitivity analysis reclassifying borderline cases (SBP 135–145 mmHg or DBP 85–95 mmHg, n=412) as non-hypertensive yielded adjusted OR=0.71 (95% CI: 0.52–0.97, p=0.031) for moderate physical activity, consistent with primary findings.

**Supplementary Section 6. Precision of Effect Estimates and Statistical Power**

Rather than relying solely on post-hoc power calculations, we emphasise the precision of our effect estimates as reflected in the 95% confidence intervals. For the primary finding of moderate physical activity’s protective association (adjusted OR=0.74, 95% CI: 0.56–0.99), the confidence interval excludes 1.0 and spans a clinically meaningful range, supporting the reliability of this estimate.

For context, with a sample size of 4,436 participants and hypertension prevalence of 40.4%, the study achieved >99% power to detect an odds ratio of 1.5 for physical activity effects and >95% power to detect an odds ratio of 1.3, indicating adequate statistical power for the primary research questions. For exposures with 25% prevalence (corresponding to HIV-positive status), power to detect OR=1.3 was 82.4% and for OR=1.5 was 98.7%.

# Supplementary Section 7. Assessment of Potential Biases

**7.1 Selection Bias**

Comparison of included and excluded participants revealed no statistically significant differences (Supplementary Table S3), suggesting minimal selection bias from missing data exclusions. However, the use of a 2009 sampling frame for a 2022–2023 survey may introduce selection bias owing to population mobility and mortality over the intervening period. Prevalence estimates should therefore be interpreted in this context rather than as population-representative figures.

**7.2 Information Bias**

Physical Activity Measurement: Self-reported physical activity via the GPAQ is subject to recall and social desirability biases. Systematic validation studies of the GPAQ suggest moderate correlation with accelerometer-based measures (r=0.45–0.65 for moderate-to-vigorous physical activity) [25,26]. Misclassification of physical activity levels would likely be non-differential with respect to hypertension status, potentially biasing associations towards the null.

Blood Pressure Measurement: Multiple automated wrist-based measurements using standardised protocols minimise measurement error. However, single-occasion measurements may misclassify some individuals, and wrist-based devices may differ from upper-arm measurements. Both sources of error would likely attenuate rather than inflate observed associations [47].

**7.3 Confounding**

Whilst we adjusted for numerous potential confounders, residual confounding from unmeasured variables (dietary sodium intake, psychosocial stress, genetic predisposition) may persist. Critically, we lacked data on antiretroviral therapy regimens, duration, and adherence, as well as CD4 count and viral load, precluding examination of therapy-specific associations amongst HIV-positive participants. This limits our interpretation of the HIV–hypertension association to overall serostatus rather than treatment-specific effects.

**7.4 Reverse Causation**

The cross-sectional design precludes determination of temporal sequence. However, the protective associations observed for moderate physical activity align with prospective cohort study findings, suggesting our results likely reflect true protective effects rather than reverse causation.

**Supplementary Table S5. Summary of Key Findings Across All Analyses**

| **Analysis** | **Moderate PA aOR (95% CI)** | **P-value** | **n** | **Consistency** |
| --- | --- | --- | --- | --- |
| Primary analysis | 0.74 (0.56–0.99) | 0.043 | 4,436 | — |
| HIV-negative only | 0.72 (0.53–0.98) | 0.037 | 3,327 | Yes |
| HIV-positive only | 0.79 (0.48–1.29) | 0.342 | 1,108 | Consistent direction |
| Excluding medication users | 0.73 (0.54–0.99) | 0.041 | 4,094 | Yes |
| Alt. BP definition (no meds) | 0.73 (0.54–0.99) | 0.041 | 4,094 | Yes |
| Borderline reclassification | 0.71 (0.52–0.97) | 0.031 | 4,436 | Yes |

*PA = physical activity; aOR = adjusted odds ratio; CI = confidence interval; BP = blood pressure. All models adjusted for the same set of covariates as the primary analysis (Table 6 in main manuscript).*

# Supplementary Section 8. Conclusions

These supplementary analyses demonstrate that the study employed appropriate statistical methods with rigorous model diagnostics and generated findings robust to multiple sensitivity analyses. The explicit documentation of the physical activity instrument (GPAQ), blood pressure measurement protocol, missing data assessment, and borderline case reclassification analysis provides the methodological transparency necessary for critical appraisal. Whilst limitations inherent to cross-sectional observational studies persist, the consistency of findings across analytical approaches, the biological plausibility of observed associations, and alignment with prospective study findings support the validity and reliability of reported associations between physical activity patterns and hypertension prevalence in this rural South African population.
