# Supplementary material for: Physical activity and hypertension amongst HIV-positive and HIV-negative populations in rural South Africa
Source: Hypertens Res. 2026 May 7;49(7):2128–40. doi: 10.1038/s41440-026-02652-2 (PMC13333498; doi:10.1038/s41440-026-02652-2)
Supplement: Supplementary file 2 — STROBE Checklist – Cross-sectional Study [file 41440_2026_2652_MOESM2_ESM.docx]

# STROBE Checklist – Cross-sectional Study

Manuscript: Physical Activity and Hypertension amongst HIV-Positive and HIV-Negative Populations in Rural South Africa

Indicate where each STROBE item is addressed in the manuscript (section/heading/table/figure).

| Item | STROBE recommendation | Location in manuscript |
| --- | --- | --- |
| 1a | Indicate the study’s design with a commonly used term in the title or the abstract. | Title; Abstract (first sentence: secondary analysis of cross-sectional survey data). |
| 1b | Provide in the abstract an informative and balanced summary of what was done and what was found. | Abstract (data source AHDSS, N=4,436, exposure GPAQ, BP device, main results incl. aORs). |
| 2 | Explain the scientific background and rationale for the investigation being reported. | Introduction. |
| 3 | State specific objectives, including any prespecified hypotheses. | Introduction (hypothesis paragraph). |
| 4 | Present key elements of study design early in the paper. | Methods – Study Design and Setting. |
| 5 | Describe the setting, locations, and relevant dates (including periods of recruitment, exposure, follow-up, and data collection). | Methods – Study Design and Setting (Aug 2022–May 2023; AHDSS location). |
| 6a | Give the eligibility criteria, and the sources and methods of selection of participants. | Methods – Study Population and Sampling (age ≥15; permanent resident; sampling frame; visits). |
| 6b | For matched studies, give matching criteria and number of exposed and unexposed. | Not applicable (no matching). |
| 7 | Clearly define all outcomes, exposures, predictors, potential confounders, and effect modifiers. Give diagnostic criteria, if applicable. | Methods – BP Measurement and Classification; Physical Activity Assessment; Assessment of Potential Confounders; Statistical Analysis (interaction terms). |
| 8* | For each variable of interest, give sources of data and details of methods of assessment (measurement). Describe comparability of assessment methods if there is more than one group. | Methods – Data Collection Procedures; Physical Activity Assessment (GPAQ/WHO STEPS); BP protocol (OMRON R6 HEM-6221-E); HIV ascertainment; Supplementary Section 2. |
| 9 | Describe any efforts to address potential sources of bias. | Supplementary – Assessment of Potential Biases; Methods/Limitations (self-report PA; wrist BP; single-visit). |
| 10 | Explain how the study size was arrived at. | Methods – Study Population and Sampling (N=4,436; exclusions described); Supplementary – Precision/Power section. |
| 11 | Explain how quantitative variables were handled in the analyses. If applicable, describe which groupings were chosen and why. | Methods – Physical Activity (MET-min/week; quintiles; low/moderate/high); BMI categories; age groups; Tables 2 & Supplementary Table S2. |
| 12a | Describe all statistical methods, including those used to control for confounding. | Methods – Statistical Analysis (logistic regression; covariate selection; adjustment set). |
| 12b | Describe any methods used to examine subgroups and interactions. | Methods – Statistical Analysis (HIV×PA interaction tested); Supplementary – stratified analyses by HIV status. |
| 12c | Explain how missing data were addressed. | Methods – Statistical Analysis (complete case; exclusions); Supplementary Section 3 (MCAR test; included vs excluded). |
| 12d | If applicable, describe analytical methods taking account of sampling strategy. | Methods – Study Population and Sampling; Limitations (no weights; 2009 frame; interpret prevalence cautiously). |
| 12e | Describe any sensitivity analyses. | Supplementary Section 5 (alternative BP definitions; exclude meds; borderline reclassification; HIV-stratified). |
| 13a | Report numbers of individuals at each stage of study (eg, numbers potentially eligible, examined for eligibility, confirmed eligible, included, completing follow-up, analysed). | Methods – Statistical Analysis (initial N=4,504; excluded 68; analysed N=4,436); Supplementary Table S3. |
| 13b | Give reasons for non-participation at each stage. | Methods – Study Population and Sampling (up to three visits); Methods – Statistical Analysis (excluded due to missing data >20%). |
| 13c | Consider use of a flow diagram. | Not included (optional). |
| 14a | Give characteristics of study participants (eg, demographic, clinical, social) and information on exposures and potential confounders. | Results – Table 1; Table 2; Table 3; Table 4. |
| 14b | Indicate number of participants with missing data for each variable of interest. | Supplementary Section 3 (missing data assessment; exclusions); Table footnotes as applicable. |
| 15 | Report numbers of outcome events or summary measures. | Results – HTN prevalence overall and by strata (Tables 1–4). |
| 16a | Give unadjusted estimates and, if applicable, confounder-adjusted estimates and their precision (eg, 95% CI). Make clear which confounders were adjusted for and why they were included. | Results – Tables 5–6; Methods – Statistical Analysis (adjustment set; covariate selection). |
| 16b | Report category boundaries when continuous variables were categorized. | Methods (BMI categories; PA cut-points; age groups); Table 2. |
| 16c | If relevant, consider translating estimates of relative risk into absolute risk for meaningful time period. | Not applicable (cross-sectional ORs; no time-to-event). |
| 17 | Report other analyses done (eg, analyses of subgroups and interactions, and sensitivity analyses). | Supplementary Section 5 (HIV-stratified; alternative BP definitions; borderline reclassification). |
| 18 | Summarise key results with reference to study objectives. | Discussion – opening paragraphs; Conclusions. |
| 19 | Discuss limitations of the study, taking into account sources of potential bias or imprecision. Discuss both direction and magnitude of potential bias. | Discussion – Study Limitations; Supplementary – Bias assessment (self-report; wrist BP; single-visit; no weights; residual confounding; ART data absence). |
| 20 | Give a cautious overall interpretation of results considering objectives, limitations, multiplicity of analyses, results from similar studies, and other relevant evidence. | Discussion (comparison with AWI-Gen/Bigna/Davis; cautious interpretation; reverse causation). |
| 21 | Discuss the generalisability (external validity) of the study results. | Limitations (single rural site; no weights; 2009 sampling frame). |
| 22 | Give the source of funding and the role of the funders for the present study and, if applicable, for the original study. | Declarations – Source of Funding. |

*STROBE item 8 applies to each variable (outcome, exposure, confounders).
